# Inositol Supplementation in the Prevention of Gestational Diabetes Mellitus

**DOI:** 10.7759/cureus.5671

**Published:** 2019-09-16

**Authors:** Faryal Tahir, Zainab Majid

**Affiliations:** 1 Internal Medicine, Dow University of Health Sciences, Karachi, PAK

**Keywords:** diabetes, gestational diabetes mellitus, inositol, myo-inositol, d-chiro-inositol, insulin resistance

## Abstract

Inositol, an emerging novel therapy for the treatment of gestational diabetes mellitus (GDM), is a cyclic polyol that has insulin-like effects and plays an important role in glucose homeostasis. The conventional treatment of GDM with insulin and oral antihyperglycemic drugs usually comes with side effects, paving the way for and shedding spotlight on clinical trials involving inositol. This review analyzed a host of recent trials that involved inositol supplementation for preventing GDM and their positive outcomes in reducing the rate of GDM among obese and overweight pregnant women, as well as women with polycystic ovarian syndrome (PCOS) or a family history of type 2 diabetes mellitus.

## Introduction and background

Gestational diabetes mellitus (GDM), which affects 14% of pregnancies globally, is defined as glucose intolerance that is first detected during pregnancy. The condition is not akin to overt diabetes, but it markedly increases the risk for the development of type 2 diabetes later on in life [1-4]. In a pregnant woman, the insulin receptors are modified due to the presence of human placental lactogen (hPL) released by the fetus, resulting in maternal hyperglycemia [5]. Moreover, a preexisting history of chronic insulin resistance can cause impaired glucose tolerance secondary to pancreatic β-cell dysfunction and subsequent hyperglycemia [6]. This leads to the excessive circulation of glucose to fetal blood, causing over-nutrition [7] along with many other complications. The diagnosis of GDM is confirmed by performing the oral glucose tolerance test (OGTT) between 24-28 weeks of gestation if an initial 1-hour oral glucose challenge test (OGCT) shows an abnormal screening result [5].

Several strategies exist for the management of GDM, including pharmacological and non-pharmacological measures. However, none of it has generated any promising or long-term results so far [1]. Currently, the recommended first step of management includes lifestyle and dietary modifications and, if non-pharmacological intervention proves insufficient, administering oral antihyperglycemic agents or insulin [7]. Insulin, the most well-known therapy for GDM, is known to cause various side effects. Its cumbersome administration method and the high chance of side effects such as hypoglycemia and weight gain have made insulin a problematic and debatable option [2].

Inositol (1,2,3,4,5,6-hexahydroxycyclohexane), which is being considered as an emerging novel intervention for GDM, is a cyclic polyol found naturally in plants and animals. It mediates cell-signal transduction along with expressing insulin-like effects [2,8]. Inositol serves as a precursor to two crucial compounds: myo-inositol (myo-Ins) and D-chiro-inositol (D-chiro-Ins), collectively known as inositolphosphoglycans (IPGs), which play an important role in the pathogenesis of diabetes [9]. Normally, insulin binds to its receptors on the cell membrane to form the insulin-receptor substrate (IRS), which in turn increases the glucose transporter type 4 (GLUT-4) translocation via the phosphatidylinositide 3-kinase (PI3K) pathway, thereby mediating glucose uptake [9]. Myo-Ins contributes to glucose homeostasis at the cellular level by activating the PI3K pathway whereas D-chiro-Ins produces secondary messengers responsible for glycogen synthesis [9]. It has been observed that conditions resulting in insulin resistance are characterized by a high level of urinary inositol metabolites [8], pointing to their efficacy as insulin mediators.

## Review

Myo-Ins supplementation in at-risk pregnant women

In a prospective, double-blind, randomized controlled pilot study involving nonobese, singleton pregnant women with an elevated fasting blood glucose of ≥92 mg/dL in their first or early second trimesters, Matarrelli et al. (2013) [10] allocated 35 women to receive 4,000 mg of myo-Ins plus 400 µg of folic acid per day throughout their pregnancies. In the same study, 38 women were placed in the placebo group and were only given 400 µg of folic acid per day for a similar duration. The diagnosis of GDM was made based on a 75-g OGTT performed at 24-28 weeks' gestation using the International Association of the Diabetes and Pregnancy Study Groups (IADPSG) criteria. The incidence of GDM was remarkably reduced and found to be only 6% in the group treated with myo-Ins when compared to the placebo group, which had a GDM-incidence rate of 71% [10]. Moreover, the group treated with myo-Ins showed remarkable improvement in terms of secondary outcomes as well: they required less insulin therapy, showed reduced incidents of premature births, and delivered significantly smaller babies with fewer instances of neonatal hypoglycemia [10].

Myo-Ins supplementation in overweight pregnant women (BMI between ≥ 25 and < 30 kg/m^2^)

In an open-label, randomized trial performed to evaluate the benefits of myo-Ins supplementation in preventing GDM among overweight pregnant women, Santamaria et al. (2015) [11] administered 2 g of myo-Ins plus 200 µg of folic acid twice a day (BD) to 95 overweight but nonobese pregnant women (pre-pregnancy BMI between ≥ 25 and < 30 kg/m^2^) from first trimester up until delivery. Similarly, a placebo group of 102 women was only given 200 µg of folic acid BD. The incidence of GDM was remarkably reduced and found to be only 11.6% in the group treated with myo-Ins when compared to the placebo group, which had a GDM-incidence rate of 27.4% [11]. Evidently, myo-Ins supplementation initiated from early pregnancy was associated with a 67% reduced risk of developing GDM in overweight but nonobese women [11]. However, there were no significant differences in terms of secondary outcomes between the two groups, such as the percentage of C-sections, infant weight and size, gestational age at delivery, shoulder dystocia, neonatal hypoglycemia, and neonatal intensive-care-unit (NICU) transfers [11].

Myo-Ins supplementation in overweight/obese pregnant women (BMI of >27 kg/m^2^)

In light of the hypothesis that overweight/obese women could benefit from myo-Ins supplementation in reducing the risk of GDM, Facchinetti F et al. (2013) [12] conducted a small randomized control trial (RCT) in which 31 singleton pregnant women with BMI of >27 kg/m^2^ were treated with 2 g of myo-Ins plus 0.2 g of folic acid BD from their first antenatal visit till delivery. None of them had abnormal glucose or glycosylated hemoglobin, any chronic disorder, or a previous history of GDM. Simultaneously, a placebo group of 40 such women was allocated to receive only 0.2 g of folic acid BD for the same duration [12]. Simple diet counseling was offered to each woman. The development of GDM was confirmed through a 75-g OGTT performed between the 24th and 26th gestational weeks. An additional OGTT was done between the 16th and 18th gestational weeks. GDM incidence was found to be 19.4% in the group treated with myo-Ins versus 40.0% in the placebo group [12]. This led the authors to conclude that myo-Ins supplementation from early pregnancy minimizes the risk of GDM in overweight women [12].

Myo-Ins supplementation in obese pregnant women (BMI of >30 kg/m^2^)

In an open-label, randomized trial, D’Anna et al. (2015) [13] treated 97 pregnant obese women (BMI of >30 kg/m^2^ before pregnancy) with 2 g of myo-Ins plus 200 µg of folic acid BD from the first trimester till delivery. A placebo group comprising another 104 pregnant obese women received 200 µg of folic acid only BD for the same period. The incidence of GDM was remarkably reduced and found to be only 14% in the group treated with myo-Ins when compared to the placebo group, which had a GDM-incidence rate of 33.6% [13], demonstrating a 66% reduced risk of GDM in the group treated with myo-Ins [13]. Moreover, a significant decrease in insulin resistance was observed in the treated group as compared to the placebo group [13]. A change in the homeostatic model assessment of insulin resistance (HOMA-IR) of -1.0 ± 3.1 was observed in the myo-Ins group as compared to that of 0.1 ± 1.8 in the placebo group [13]. Therefore, it was concluded that supplementation with myo-Ins could reduce the rate of GDM in obese women by improving their insulin sensitivity [13].

Myo-Ins supplementation in pregnant women with a family history of diabetes

In an open-label, randomized study conducted on Caucasian women, D’Anna et al. (2013) [14] treated 99 pregnant women who had a parent with type 2 diabetes. They were given 2 g of myo-Ins plus 200 µg of folic acid BD while a placebo group of another 98 women was given only 200 µg of folic acid BD from the first trimester till delivery. GDM was diagnosed based on IADPSG criteria. The incidence rate of GDM in the group treated with myo-Ins was found to be 6% as compared to 15.3% in the placebo group [14], showing a 65% reduction in the risk for GDM in the group treated with myo-Ins [14]. Moreover, a significant decline in fetal macrosomia and birth weight was highlighted in the intervention group [14].

Farren et al. conducted another RCT involving women in early pregnancy with a family history of diabetes (2017) [8]. In this study, the researchers employed a combination therapy involving myo-Ins and D-Chiro-Ins. They treated 120 women with a daily dose of 1,100 mg of myo-Ins plus 27.6 g of D-Chiro-Ins along with 400 µg of folic acid. A placebo group of another 120 women was given a daily dose of 400 µg of folic acid only. OGTT was performed on all women between 24 and 28 weeks of gestation. The incidence of GDM was noted to be 23.3% in the group treated with myo-Ins, while the placebo group showed a GDM incidence rate of 18.3% [8]. These findings led the authors to conclude that a combination therapy with myo-Ins and D-Chiro-Ins, even when commenced in the early pregnancy, failed to prevent GDM in women with a family history of diabetes [8].

Myo-Ins supplementation in pregnant women with polycystic ovarian syndrome (PCOS)

In a retrospective study, D’Anna et al. (2012) [15] treated 46 anovulatory and hyperinsulinemic non-diabetic women with polycystic ovarian syndrome (PCOS) by administering 4 g of myo-Ins plus 400 µg of folic acid BD from preconception till delivery. Simultaneously, a placebo group of 37 such women was given 1.5 mg of metformin plus 400 µg of folic acid BD from preconception up until a positive pregnancy test. A significant difference in the prevalence of GDM was noticed between the groups, with the group treated with myo-Ins showing a GDM-incidence rate of 17.4% as compared to 54% in the placebo group [15]. The risk of GDM incidence was found to be more than two-fold in the placebo group [15], clearly illustrating the positive effects of myo-Ins in the primary prevention of GDM among women with PCOS [15]. However, there were no significant differences in terms of secondary outcomes between the two groups, such as hypertensive disorders, C-sections, and premature or macrosomic babies [15].

Myo-Ins supplementation in patients with diagnosed GDM

In a study conducted among diet-treated Caucasian patients with diagnosed GDM, Corrado et al. (2011) [16] treated a study group consisting of 24 women with 4 g of myo-Ins plus 400 µg of folic acid daily. A placebo group comprising 45 women received a daily dose of 400 µg of folic acid only. Following eight weeks of treatment, a 50% reduction in the HOMA-IR was found in the study group compared to 29% in the placebo group [16], establishing that myo-Ins supplementation leads to an improvement in insulin resistance in patients with diagnosed GDM [16].

Recently, Vitagliano A et al. [17] and Guo X et al. [18] conducted two systemic reviews and meta-analysis on the aspect of myo-Ins supplementation in pregnant women. Vitagliano A et al. [17] conducted five RCTs with 965 participants and concluded that myo-Ins administration during pregnancy can be a safe and novel strategy to prevent GDM. In particular, they found that the administration of 2 g of myo-Ins BD may improve glycemic homeostasis and ultimately reduce the GDM rate. Guo X et al. [18] performed four RCTs with 586 participants and concluded that myo-Ins lowers the incidence of GDM along with a reduction in the fasting, 1-hour, and 2-hour OGTT values during pregnancy.

The table below (Table 1) summarizes the RCTs discussed in our review.

**Table 1:** Summary of randomized control trials (RCTs) discussed in our review GDM: gestational diabetes mellitus; BMI: body mass index; PCOS: polycystic ovarian syndrome

Authors	Year of study	Population	Incidence of GDM (%)	
			Study group	Placebo group
Matarrelli et al. [10]	2013	At risk for GDM	6	71
Santamaria et al. [11]	2015	Overweight (BMI between ≥ 25 and < 30 kg/m^2^)	11.6	27.4
Facchinetti F et al. [12]	2013	Overweight (BMI of >27 kg/m^2^)	19.4	40.0
D’Anna et al. [13]	2015	Obese (BMI of >30kg/m^2^)	14	33.6
D’Anna et al. [14]	2013	Family history of diabetes	6	15.3
Farren et al. [8]	2017	Family history of diabetes (combination therapy of myo-Ins and D-Chiro-Ins)	23.3	18.3
D’Anna et al. [15]	2012	PCOS	17.4	54

Safety and dosage of Inositol during pregnancy

Myo-Ins is currently sold in the US as a medium to promote healthy ovarian function among women planning to get pregnant. Even after so many years of over-the-counter use, no serious side effects have been noted. No adverse events have been reported in the literature reviews of any of the RCTs in which myo-Ins was given to pregnant women. In fact, many of the trials reported a good tolerance to the drug [10-11,13-16,19-23]. Beginning with the 1980s, it was suspected that some cases of delayed lung development in the fetus could be a consequence of maternal inositol supplementation as it causes increased production of IPGs at the expense of phosphatidylglycerol. However, later animal studies failed to confirm this hypothesis [24-27]. Studies have shown that a dose of up to 4 g of inositol per day has been well-tolerated by pregnant women without any adverse effects [10,21], though more research is required before the routine use of the supplement can be recommended. However, 2 g of myo-Ins with 400 µg of folic acid DB has been routinely recommended for blood-sugar control in patients with GDM [10,14,21].

## Conclusions

A cumbersome administration method and a high chance of side effects such as hypoglycemia and weight gain have made insulin a problematic and debatable option in the prevention of GDM. However, the nutrition supplement inositol has been shown to be a safe and easy-to-administer alternative. On the basis of current evidence, myo-Ins supplementation reduces the risk of GDM among obese and overweight pregnant women, as well as women with PCOS or a family history of type 2 diabetes mellitus. However, this conclusion needs to be further evaluated in large-scale, multi-center, double-blinded RCTs before inositol administration can be recommended as a routine treatment paradigm for the prevention of GDM.
